# The Use of Ultrasound Imaging in the External Beam Radiotherapy Workflow of Prostate Cancer Patients

**DOI:** 10.1155/2018/7569590

**Published:** 2018-01-24

**Authors:** Saskia M. Camps, Davide Fontanarosa, Peter H. N. de With, Frank Verhaegen, Ben G. L. Vanneste

**Affiliations:** ^1^Faculty of Electrical Engineering, University of Technology Eindhoven, Eindhoven, Netherlands; ^2^Oncology Solutions Department, Philips Research, Eindhoven, Netherlands; ^3^School of Clinical Sciences, Queensland University of Technology, Brisbane, QLD, Australia; ^4^Institute of Health & Biomedical Innovation, Queensland University of Technology, Brisbane, QLD, Australia; ^5^Department of Radiation Oncology (MAASTRO), GROW School for Oncology and Developmental Biology, Maastricht, Netherlands

## Abstract

External beam radiotherapy (EBRT) is one of the curative treatment options for prostate cancer patients. The aim of this treatment option is to irradiate tumor tissue, while sparing normal tissue as much as possible. Frequent imaging during the course of the treatment (image guided radiotherapy) allows for determination of the location and shape of the prostate (target) and of the organs at risk. This information is used to increase accuracy in radiation dose delivery resulting in better tumor control and lower toxicity. Ultrasound imaging is harmless for the patient, it is cost-effective, and it allows for real-time volumetric organ tracking. For these reasons, it is an ideal technique for image guidance during EBRT workflows. Review papers have been published in which the use of ultrasound imaging in EBRT workflows for different cancer sites (prostate, breast, etc.) was extensively covered. This new review paper aims at providing the readers with an update on the current status for prostate cancer ultrasound guided EBRT treatments.

## 1. Introduction

Prostate cancer is the most frequently diagnosed cancer in men worldwide. It accounted for 1.6 million new diagnoses and 366,000 deaths in 2015 [1]. In the next decades, the incidence of prostate cancer might increase due to the possible linkage of this cancer with risk factors associated with economic development (e.g., excess body weight and physical inactivity) [2] and the aging population [3].

One of the curative treatment modalities for prostate cancer is external beam radiotherapy (EBRT) [3]. The aim of this modality is to irradiate tumor tissue using ionizing radiation generated by an X-ray source (e.g., linear accelerator). At the same time, normal tissue must be spared as much as possible to avoid excessive toxicity. EBRT is one of the most common forms of RT treatment and therefore it is often denoted as just radiotherapy (RT) in literature (as will be done in the remainder of this paper).

Prior research using kV radiography has shown [4, 5] that frequent imaging of the patients' anatomical structures of interest during the course of the prostate RT treatment (image guided RT, IGRT) can improve radiation targeting and tumor control. This improved targeting could allow reduction of safety margins, with consequently decreased toxicity. Next to kV radiographs also other imaging modalities have been used for IGRT, such as cone beam CT (CBCT) in combination with fiducial markers [6], magnetic resonance imaging (MRI) [7], implantation of electromagnetic transponders [8], and ultrasound (US) imaging [9].

In this review paper the focus solely lies on the use of US imaging during the IGRT workflow of prostate cancer patients. US imaging typically provides good soft-tissue contrast and therefore it is a modality that allows contouring of structures such as the prostate [10]. It is also a real-time image modality, because the images are reconstructed and visualized directly during the acquisition. Some of the currently available US systems potentially even allow real-time volumetric imaging and soft-tissue tracking, using a matrix probe (e.g., X6-1 xMatrix array probe, center frequency: 3.2 MHz, Philips Healthcare, Bothell, WA, United States), or a mechanically swept probe (e.g., Clarity Autoscan probe, m4DC7-3/40, center frequency: 5 MHz, Sonix Series; Ultrasonix Medical Corporation, Richmond, BC, Canada).

Some of the limitations and challenges associated with US imaging include the inaccessibility of tissue shielded by bone or air, the proneness for imaging artifacts, and the user dependency [17], due to its mostly manual operation. However, in comparison with other imaging modalities US is cost-effective and it does not deliver ionizing radiation to the patient. The combination of these characteristics with the real-time volumetric tracking ability makes US imaging a suitable image modality for inter- and intrafraction organ motion monitoring during the course of a prostate RT treatment [34]. US imaging could then be used either as standalone system or possibly in combination with other imaging modalities.

In 2015 and 2016 two review articles [11, 12] were published in which the use of US for IGRT of different cancer sites (e.g., prostate, breast, and liver) was extensively covered. The current review article updates this work for prostate cancer. After an introductory summary on US techniques and US systems that can potentially be used during the RT prostate cancer patient workflow, a comprehensive update on the latest developments in this field is presented. 

## 2. EBRT Workflow and US Imaging

### 2.1. EBRT Workflow

**Figure 1:**
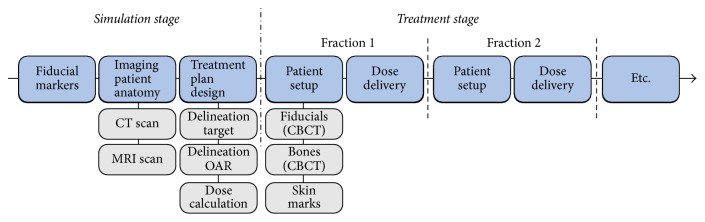
Typical RT workflow for prostate cancer patients. During the simulation stage, fiducial markers are implanted in the prostate, images of the patient's anatomy are acquired, and a treatment plan is designed. Subsequently, the dose is delivered to the patient in several treatment fractions, while ensuring that the patient is set up as accurately as possible.

The typical RT workflow of prostate cancer patients consists of several steps, belonging to either the simulation stage (preparatory phase) or the treatment stage (radiation dose delivery phase) (Figure 1). The first step involves the invasive implantation of fiducial markers in the prostate gland. These markers are considered a surrogate for the target and are currently used to monitor its motion between different treatment fractions using X-ray imaging.

Subsequently, a computed tomography (CT) scan and increasingly more often an MRI scan are acquired. The CT scan provides electron-density information allowing for treatment plan preparation, based on prescribed radiation dose and delineations of the anatomical structures of interest (target and organs at risk [OARs]). In case also an MRI is acquired, it is registered with the CT scan based on the fiducial markers [35], which can be visualized with both imaging modalities. Then, the prostate (target) is delineated on the MRI instead of on the CT scan. As the volumes are registered, the delineation can be transferred to the CT scan and used during the treatment plan preparation. MRI-based delineation is preferred as MRI usually allows for a more accurate delineation of the prostate than the CT [36–38].

After finalizing the treatment plan design, the radiation dose will be delivered to the patient in multiple daily treatment fractions (up to 45) during 1-2 months [3]. The setup of the patient prior to each of these treatment fractions is an important step in the RT workflow. This procedure must be as accurate as possible to reproduce the setup at simulation stage, on which the treatment plan was designed.

Nowadays, setting up the patient is typically assisted by the use of skin marks on the patient's body [39], the previously mentioned fiducial markers [40], and CBCT [41]. However, even if the patient seems to be correctly aligned, internal soft-tissue deformations may still occur. The position and shape of the prostate can change, due to a different filling of the bladder and rectum [42]. To account for these deviations from the simulation CT, a safety margin is usually added to the treatment target [18]. Unfortunately, this leads to a larger volume being irradiated, potentially including larger portions of OARs.

Monitoring the position and shape of the prostate during the course of the RT treatment could potentially improve the accuracy of the radiation dose delivery and, in the end, potentially even allow for a margin reduction. In the ideal case, this prostate monitoring would not only include monitoring between different fractions (interfraction), but also during a treatment fraction (intrafraction) [12]. As noted before, US imaging could be a suitable imaging modality for this purpose.

### 2.2. US Imaging in RT Workflow

US imaging makes use of a probe equipped with piezoelectric elements to create high-frequency sound waves and transmit these into the body. On their way through the body, these waves encounter interfaces between different tissues and scattering objects. Due to the difference in acoustic impedance between the tissues at each side of this interface and between the scattering objects and the surrounding tissue, a part of the US waves is reflected, while the remaining waves keep penetrating deeper into the body. The reflected waves are received by the probe, processed, and combined to generate an image.

As air reflects US waves very strongly, the presence of air between the probe and the body of the patient will prevent sufficient penetration of the waves into the body, which significantly degrades the image quality. It is therefore crucial to establish sufficient acoustic coupling between the probe and the body. For this purpose, a coupling medium, such as US gel or water, is typically used.

Several US probes with different shapes and characteristics are commercially available for the different procedures possible with this technology. To image the prostate and OARs during the RT workflow, three US imaging techniques are presently used in clinical practice. These techniques and how they can potentially improve the accuracy of radiation dose delivery are described in the next sections. We refer to the literature (e.g., [43, 44]) for more general details on the physics theory and technology of US imaging.

#### 2.2.1. Transrectal US Imaging

**Figure 2:**
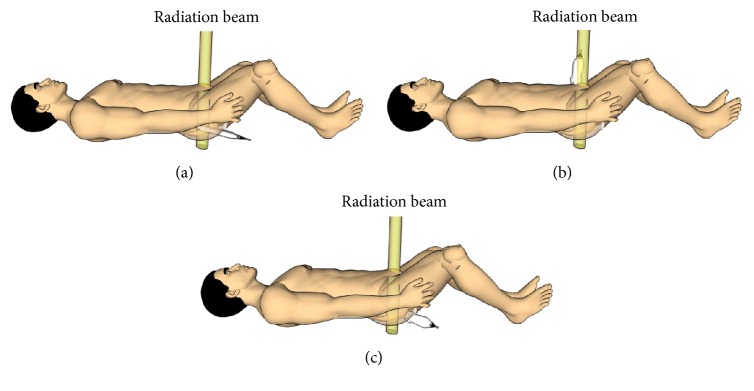
US probe setup using three US imaging techniques. (a) TRUS, (b) TAUS, and (c) TPUS with the yellow beam indicating a possible location of a radiation beam during a treatment fraction.

**Figure 3:**
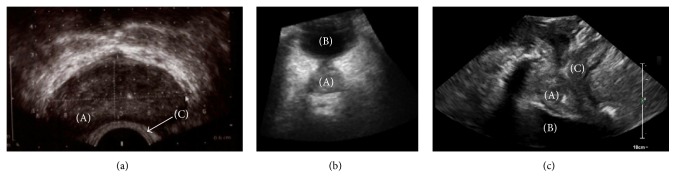
Three US techniques suitable for prostate and OARs imaging (a) TRUS, (b) TAUS, and (c) TPUS, with (A) prostate, (B) bladder, and (C) rectum which can partially be seen.

Transrectal US (TRUS) imaging requires positioning of the probe through the anus inside the rectum (Figure 2(a)) and is therefore a low invasive imaging procedure. As the prostate is located in close proximity of the rectum, TRUS allows imaging of the prostate with a good image quality (Figure 3(a)) [45]. Challenges that can occur while making use of TRUS imaging are rectal filling, which can be removed using an enema [46], and the potential presence of air in the rectum, which results in a poor acoustic coupling between the probe and the body of the patient.

**Figure 4:**
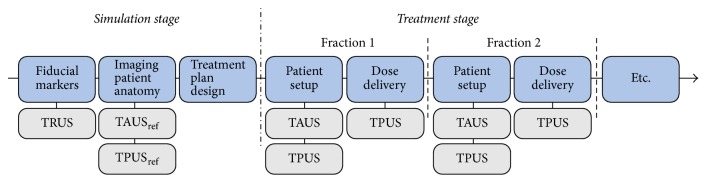
RT workflow of prostate cancer patients with US imaging implemented at several steps. The fiducial marker implantation is currently performed under TRUS guidance. The acquisition of the reference TAUS or TPUS images at simulation stage and also the acquisition of TAUS and TPUS prior to dose delivery can provide valuable information for interfraction prostate motion correction. Finally, during dose delivery TPUS imaging could provide information on intrafraction prostate motion.

In the EBRT workflow, TRUS imaging is currently used to the guide fiducial marker placement during the simulation stage (Figure 4) [47]. The invasive character of this US modality makes it less suitable for frequent imaging during the course of the treatment. In addition, the presence of the probe inside the rectum being potentially in the path of the radiation treatment beam (Figure 2(a)) raises issues as well. For this reason, no research seems to have been conducted on the use of TRUS for inter- and intrafraction organ motion monitoring during prostate EBRT.

#### 2.2.2. Transabdominal US Imaging

Transabdominal US (TAUS) imaging involves the positioning of the US probe on the abdomen (Figure 2(b)) and it is therefore a noninvasive imaging modality. It is capable of measuring the same prostate volumes as TRUS imaging (considered the standard) [48] and it makes use of the acoustic window of the bladder for prostate visualization (Figure 3(b)). For this reason, TAUS requires a reasonably full bladder, which might lead to discomfort for the patients. However, a filled bladder is often requested during the RT treatment to prevent the whole bladder wall from being irradiated and to push the intestines away from the high dose regions.

During TAUS imaging, the probe is located relatively far from the prostate, which might influence the quality of the acquired images. Particularly the acquisition of TAUS images of obese patients is a challenge [49]. Adipose tissue attenuates the US waves and increases the possibility for imaging artifacts, which can significantly degrade the image quality. Unfortunately, it is a challenge to predict the degree of adipose attenuation and the associated image quality degradation, due to the dependence on patient-specific characteristics, such as fat distribution [50].

The probe setup on the body of the patient during TAUS imaging makes this imaging modality suitable for interfraction monitoring. However, it is more challenging to use TAUS imaging for intrafraction monitoring (Figure 4), as the probe is potentially located in the path of the radiation beam, especially for rotational therapy (Figure 2(b)). Ways to overcome this challenge are currently not available in clinical practice, although they are being investigated. In Section 6 of this paper, the recent developments in this field will be discussed.

In the past 20 years, three systems were commercially available that allowed interfraction monitoring of the prostate during the RT workflow by means of TAUS imaging: SonArray system (Varian Medical Systems, Palo Alto, CA, USA), B-Mode Acquisition and Targeting (BAT) system (Best Nomos, Pittsburgh, PA, USA), and the Clarity system (Elekta, Stockholm, Sweden, formerly called Restitu and commercialized by Resonant Medical, Montreal, QC, Canada). To our knowledge, only the Clarity system is still available on the market and as there have been papers published on this system in the last years, it will be covered in this paper.

The BATCAM system was only used in one study [51] since the publication of the previously mentioned review papers [11, 12]. In this study a comparison was made between the Clarity system and the BATCAM system, resulting in a good agreement between both. As the BATCAM system was extensively covered in the previous review papers, it will not be discussed further in this work.

In the RT workflow, a freehand sweep using a 2D TAUS probe (C5-2/60, center frequency: 3.5 MHz, Sonix Series; Ultrasonix Medical Corporation, Richmond, BC, Canada) can be acquired by the Clarity system during the simulation stage. Due to the use of a probe localization system, it is possible to reconstruct the sweeps such that a 3D TAUS volume is created. The same procedure is repeated prior to each treatment fraction. The requirement for manual sweep acquisition makes the Clarity system inherently sensitive to uncertainties associated with operator variability and probe pressure. These issues will be covered in more detail in Section 5.

Comparison of the US volumes acquired at treatment stage and the reference US volume acquired at simulation stage allows the calculation and correction of interfractional prostate motion [34]. Besides the fact that the US probe is potentially located in the path of the radiation beam, the need of an operator performing the manual sweep for the 3D TAUS volume reconstruction makes this system not suitable for intrafraction monitoring.

#### 2.2.3. Transperineal US Imaging

Transperineal US (TPUS) imaging is a noninvasive imaging modality, as it involves the positioning of the US probe on the perineum of the patient (Figure 2(c)). Also this imaging modality is capable of measuring the same prostate volumes as TRUS imaging [52]. TPUS imaging does not exploit the acoustic window of the bladder to obtain images of the prostate (Figure 3(c)) and therefore it requires a less strict bladder filling protocol. A semifilled bladder is still beneficial since it yields good imaging contrast distal to the prostate. In addition, as the distance between the prostate and the perineum is smaller, a relatively good image quality can potentially be achieved. However, just like with TAUS imaging, the body composition of the patient can affect the image quality. Finally, due to the fact that the probe setup does not interfere with the radiation beam (Figure 2(c)), TPUS imaging can potentially be used also for intrafraction monitoring of the prostate (Figure 4).

Currently there is only one commercial system available that enables the inter- and intrafraction prostate motion monitoring during the RT workflow using TPUS imaging: Clarity Autoscan (Elekta, Stockholm, Sweden) [34]. This system is an extension of the Clarity system as described above. Like the Clarity system it employs a 2D probe (m4DC7-3/40, center frequency: 5 MHz, Sonix Series; Ultrasonix Medical Corporation, Richmond, BC, Canada). However, the Autoscan probe is mounted in a housing which also comprises a motorized control of the sweeping motion. This automation of the sweeping motion makes a manual sweep superfluous.

**Figure 5:**
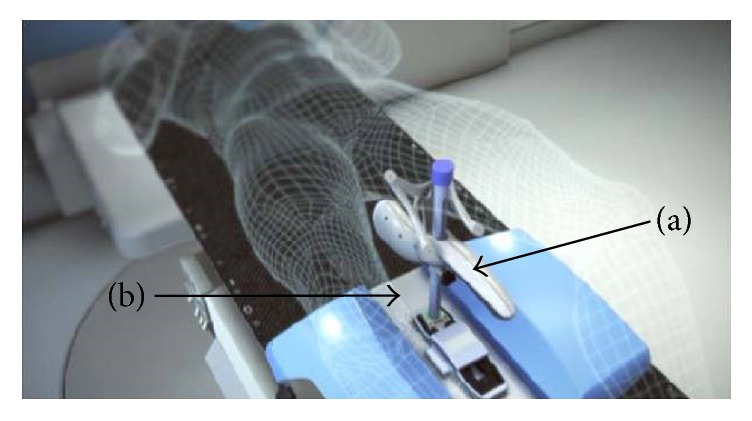
Clarity Autoscan system setup with (a) probe and (b) baseplate.

The Autoscan probe which can be localized in the room by a probe tracking system is attached to a baseplate on the CT or on the linear accelerator (LINAC) couch during the procedure (Figure 5), allowing positioning and locking of the probe for TPUS imaging. The use of the baseplate and the automatically performed sweeping motion potentially reduce the operator dependence. The operator dependence will be covered in more detail in Section 5.

The Clarity Autoscan system follows the TPUS workflow, as represented in Figure 4. First, a 3D TPUS volume is acquired at simulation stage. Then, prior to the dose delivery, a full sweep is acquired and reconstructed. Comparison of this full sweep with the image acquired at simulation allows the calculation of a required couch shift to account for interfraction prostate motion.

During the radiation dose delivery, continuous volumetric imaging using the US probe is performed. This allows position monitoring of the prostate in 3D. The therapist can interrupt the treatment and perform a couch correction, in case the motion in a certain Cartesian direction is exceeded for a certain amount of time. These motion direction and time thresholds can be set by the operator prior to the first treatment delivery [34].

## 3. Interfraction Monitoring

### 3.1. Fiducial Markers

As already introduced in Section 2, currently 3-4 fiducial markers are implanted prior to the start of the radiation treatment. The most frequently used markers are made of gold and provide a surrogate for the prostate position. The markers are visible using kV imaging modalities (such as CBCT or 2D X-ray radiographs) but can also cause metal-induced image artifacts [40].

The implantation procedure is often performed under TRUS guidance and involves invasively positioning the markers in the prostate through the perineum or the rectum [53]. The procedure can be considered as well tolerated by the majority of patients [47, 54], but it is definitely not without risks. One study [55] even suggests that the risk associated with the implantation of the markers through the rectum is still underestimated. An overall rate of symptomatic infection with the fiducial marker implantation was reported to be 7.7% with one-third requiring hospital admission.

The use of fiducial markers during the RT workflow is based on the assumption that the marker position inside the prostate will not change during the whole course of the treatment, from the simulation stage until the final treatment fraction. Changes in anatomy and physiology, however, can potentially cause or mimic marker migration [56]. Moreover, studies have shown that the presence of fiducial markers in the prostate can affect the dose deposition [57] and that imaging the fiducial markers using CBCT adds a nonnegligible dose to the patient [58].

Therefore, interfraction motion monitoring should be ideally performed with a noninvasive image modality that does not require the presence of these fiducial markers inside the prostate. In this regard, US imaging is an excellent candidate. In the next section studies are discussed which used TAUS or TPUS imaging for interfraction motion monitoring of the prostate.

### 3.2. TAUS and TPUS Imaging

In Table 1 the studies are reported that compared the use of TAUS (Clarity system) or TPUS (Clarity Autoscan system) with other imaging modalities for interfraction prostate monitoring. As the work of Tas et al. [59] only includes data from one prostate cancer patient, it is excluded from this table. The studies indicated with an asterisk (*∗*) were included in the previously mentioned review papers [11, 12]. However, they have been added to this work to provide a complete overview.

The older studies primarily focused on TAUS imaging. In these studies, 2D techniques [13, 15, 17] and volumetric imaging techniques [14, 16, 19, 20] were used for comparison with the TAUS imaging. One study [21] also compared the results of a surface imaging system (AlignRT, VisionRT, London, UK) with TAUS imaging. The four most recent studies [22–25] examined the use of TPUS imaging in comparison with volumetric imaging only, such as CBCT and an additionally acquired planning CT.

All studies (TAUS and TPUS) reported the differences (using mean ± standard deviation (SD) or error notation including mean and systematic and random error [18]) between the US imaging technique and another image modality. The reported mean differences for the anterior-posterior (AP), left-right (LR), and superior-inferior (SI) directions were in 9 out of 11 studies in the absolute range of 0–3 mm. Some studies also reported the Bland-Altman 95% limits of agreement (LoA) [60] and/or the ranges of the measured differences. For the studies that did not report the LoA, the ranges are detailed in the final column of Table 1. The largest range difference was reported by Robinson et al. [16], ranging between 1.3 mm and 61.4 mm.

The Bland-Altman LoA are detailed in the final column of Table 1 and were reported by 5 out of 12 studies. The LoA (bias ± 1.96*∗*SD) are a measure for the interchangeability of two methods or systems. If the limits are smaller than or equal to an a priori defined tolerance, one method can be used interchangeably with the other. The TPUS studies (min LoA: 3.2 mm; max LoA: 9.4 mm) tend to report slightly lower LoA values than the TAUS studies (min LoA: 5.3 mm; max LoA: 11.7 mm). Considering that the prostate safety margins currently used in clinical practice (using fiducial markers) range from 3 to 10 mm [61], neither TAUS nor TPUS could be considered interchangeable with the imaging techniques they have been compared with. However, this does not automatically imply that the US techniques perform worse than the comparison technique, simply because there is no recognized ground truth. Therefore, potential inaccuracies in the imaging modality that the US is compared with can influence the results and associated conclusions.

The absence of ground truth is also reflected in conflicting conclusions regarding the potential performance of US imaging in the RT workflow. For example, Li et al. [19] concluded that it is feasible to use TAUS imaging for image guidance during the prostate RT workflow and that this image modality appears comparable to CBCT when used for the same purpose. On the other hand, Fargier-Voiron et al. [20] concluded that TAUS imaging cannot replace CBCT without increasing treatment margins. These conclusions seem to differ significantly, while the reported mean differences between the reference imaging modality and TAUS imaging are comparable.

In general, it seems that the studies investigating the use of TPUS imaging are more optimistic about the accuracy, interchangeability, and usability in comparison to the TAUS imaging studies. For example, Trivedi et al. [24] conclude that TPUS imaging provides excellent imaging of the prostate and comparable localization results. Also Li et al. [25] conclude that TPUS is a feasible image modality for IGRT and has a good accuracy.

In conclusion, different opinions exist in the literature regarding the comparability between US (TAUS and TPUS) and other imaging modalities used for image guidance during the RT workflow. For this reason, more research is necessary before final conclusions can be drawn about the usability of US imaging in the prostate IGRT workflow. Also, it is very important that US imaging is standardized to reduce the operator dependency (see Section 5).

## 4. Intrafraction Monitoring

As discussed in the introduction section, the position and shape of the prostate can change, due to, for example, different bladder or rectum fillings. This phenomenon can occur not only between treatment fractions, but potentially also during a treatment slot. Intrafractional prostate motion has been investigated in several studies using, for example, the Calypso localization system (Calypso Medical Technologies, Inc., Seattle, WA, USA) (e.g., [8, 62]). This system is based on the electromagnetic detection of beacon transponders which need to be implanted in the prostate. Calypso provides continuous, real-time localization of the prostate surrogates and it has been shown to have a submillimeter accuracy in a phantom [63].

These transponders need to be implanted in the prostate and, in addition, can cause image artifacts on MRI that could be used for treatment response assessment. In addition, an antenna which is necessary for the localization of the beacons is present in the path of the radiation beam. Finally, assumptions are needed to determine a relation between the position of the transponders and the shape and location of the prostate, making the Calypso system not a real volumetric tracking system.

As the Clarity Autoscan system (TPUS) does not involve implantation of transponders in the prostate, it allows for real volumetric tracking of the prostate. In addition, during the procedure no equipment is present in the beam path, which potentially makes it a more favorable solution for intrafraction prostate motion tracking in comparison to the Calypso system. Abramowitz et al. [64] found a good agreement between the Clarity Autoscan system and the Calypso system, while examining the ability of both systems to track a prostate-like sphere in a phantom.

The accuracy and precision of the Clarity Autoscan system have been evaluated in a study using a male pelvic phantom [65]. In this study, a latency of 223 ± 45.2 milliseconds was reported between the motion of the phantom and the US tracking. In addition, a mean position error of 0.23 mm (LR) and 0.45 mm (SI) was reported. These positional and timing accuracies were found to be acceptable under the simulated treatment conditions examining, among others, the performance of the system while the radiation beam was on and while the image quality was degraded by the introduction of an air gap between the probe and the surface of the phantom. This was done to assess tracking performance under worse image quality conditions.

In the literature, three papers [26, 27, 66] and one abstract [28] are available in which intrafraction prostate monitoring was clinically investigated using the Clarity Autoscan system. The authors of these publications reported different metrics. For example, Richardson and Jacobs [27] reported the total frequency of intrafraction prostate displacements per direction for different thresholds, while Baker and Behrens [26] reported the percentage of fractions with displacements larger than 2 mm. These differences make it difficult to compare the results directly.

Ballhausen et al. [66] investigated data from 6 prostate cancer patients. This data was used to verify their hypothesis that the intrafraction motion of the prostate can be modeled as a time-dependent “random walk” [67]. It was shown that the prostate tends to move away from the treatment isocenter during a fraction and that this drift away from the isocenter increases over time. These findings imply that a shorter dose delivery time could be favorable. Such a reduction of the treatment time can be achieved by using, for example, volumetric modulated arc therapy (VMAT) or RapidArc® Radiotherapy Technology (see [3] for more details on radiation techniques).

Baker and Behrens [26] investigated the prostate intrafraction motion during a time interval corresponding to a beam-on time for RapidArc (120–150 seconds) (see Table 2). A tolerance of 2 mm was considered, as this value is perceived to be clinically irrelevant according to the British Ionization Radiation Medical Exposure Regulations 2000 (IRMER 2000). In the study, maximal intrafractional displacements of −0.2 ± 1.1 mm (AP), −0.2 ± 0.8 mm (LR), and +0.2 ± 0.9 mm (SI) were found. The largest displacement of 2.8 mm was measured in the posterior direction. Also, displacements of larger than 2 mm were measured for 10% (AP), 2% (LR), and 4% (SI) of the examined fractions. The authors concluded that the displacement of the prostate is insignificant during the measured time interval. However, the conclusion was also drawn that the displacement increases over time, which is in line with the findings of Ballhausen et al. [66].

Richardson and Jacobs [27] instead used the Clarity Autoscan system to assess the intrafraction prostate motion during intensity-modulated radiotherapy (IMRT) with static beams from different angles, which consequently has a longer treatment time (reported mean of 385 seconds). In this case, the authors considered three different thresholds: 3 mm (fine tolerance), 7 mm (future planning target volume), and 10 mm (current planning target volume). In addition to a technical overview, also the first clinical experiences of the physicians were captured in a letter [68] and article [69].

Also in this study, the motion of the prostate in the posterior direction seems to be the most common (Table 2). All patients experienced at least one displacement larger than 3 mm and 35% of the patients experienced one displacement larger than 10 mm. These higher rates of motion in comparison with [26] can potentially be explained by the fact that the evaluated time interval was much longer (385 seconds versus 120–150 seconds). In the study of Richardson and Jacobs [27] also the duration of the intrafraction prostate displacement was calculated as a proportion of the total treatment time. This duration varied considerably between patients. For example, for motion larger than 3 mm in the posterior direction, durations from 2% of the treatment time up to 92% of the treatment time were observed for individual patients.

Finally, also one abstract was published by Guillet et al. [28] in which the dosimetric impact of the intrafraction motion was investigated and in which also some prostate movement results were reported. Also in this work, the largest movements were reported in the AP direction (Table 2), with 18% of the short treatment sessions (140 seconds) and 31% of the longer treatment sessions (290 seconds) displaying motions larger than 3 mm. In addition, in this work it was also shown that the dosimetric impact of the intrafractional motion increases with the treatment time duration.

## 5. Operator Dependence

Currently, the operator who acquires the US images in the clinic (not only in the RT environment) may need to (manually) place the US probe on the body of the patient, interpret the live images, and then decide if the correct anatomical structures are visualized with sufficient image quality. This makes US imaging operator dependent and this dependence may cause significant variability in the quality of the acquired US images and thus influence the ability to locate and track the prostate and OARs.

Section 5.1 discusses the studies that investigated prostate displacement induced by probe pressure in both TAUS and TPUS. Inter- and intraoperator variability is detailed in Section 5.2.

### 5.1. Probe Pressure Effects

As introduced previously, the Clarity system requires the acquisition of a manual sweep along the abdomen of the patient using the TAUS probe prior to radiation dose delivery. The acquired image can then be used for interfraction motion correction. Subsequently, the probe is removed from the body of the patient and the patient is irradiated. In case the prostate is displaced due to probe pressure, it might move to a different position when the probe is removed from the body. This displacement after the probe removal is not accounted for in the interfraction motion correction, which might lead to a suboptimal radiation dose delivery.

Table 3 details studies that investigated prostate displacement due to probe pressure. Two out of three TAUS studies used a relative method to assess the prostate displacement. For example, Van Der Meer et al. [17] acquired images at no pressure (reference situation: probe touching the skin) and subsequently acquired images at low pressure, intermediate pressure, and high pressure. To determine the displacement due to probe pressure the location of the prostate was compared to the reference situation.

Baker and Behrens [30] assessed the effect of TAUS probe positioning using TPUS imaging. In this work, a reference image was acquired using just a TPUS probe without the TAUS probe actually being in place on the body of the patient. The average displacement vector of the prostate found by Baker and Behrens [30] was significantly lower than the distance found in the other studies (1.3 mm versus 2.5 mm and 3.0 mm). The studies concluded that even though the prostate displacements are small, a minimal pressure should be used in order to make the probe setup more reproducible.

The effect of probe pressure during TPUS imaging were reported in two studies. Mantel et al. [70] investigated the shift of the penile bulb after positioning the TPUS probe against the perineum. A superior shift of the penile bulb could bring it closer to the prostate and therefore closer to the high dose region. This could lead to an increase of dose delivered to the penile bulb, which has been correlated earlier (e.g., [71]) with the incidence of erectile dysfunction. The authors studied datasets from 10 patients and reported that the penile bulb had a significant median shift of 6.2 mm in the superior direction. In addition, no relevant volume changes of the prostate and planning target volume due to probe pressure were observed and just minor motion of these structures was reported, mainly in the superior direction. No quantitative results on this prostate and planning target volume motion were reported in the paper.

In another study [31] the pressure applied by a TPUS probe was found to have a quantitatively similar impact on prostate displacement as the TAUS probe (Table 3). As this conclusion contradicts the conclusion of Mantel et al. [70], it implies that more research is necessary to understand the impact of TPUS probe pressure on the displacement of the prostate and OARs. Li et al. [31] also detected a systematic intrafraction drift of the prostate. They hypothesized that this drift was caused by the relaxation of the compressed tissue of the perineal area present between the prostate and the probe. As intrafraction motion monitoring is possible using TPUS imaging, this drift can be monitored and, when needed, potentially compensated for.

With TPUS imaging the probe does not need to be removed prior to dose delivery. Therefore, no displacement of the prostate and organs at risk due to probe removal is expected. As long as the pressure is not so high that it produces a shift of the OARs into high dose regions (as reported e.g., for the penile bulb in the previous paragraph) and it is reproducible, the consequences of the pressure in the US guided RT workflow should be minimal. For TAUS imaging, it was reported that it is difficult to reproduce the pressure [29]; however, for TPUS imaging results on this issue are currently not available. If future studies prove that it is feasible to position the TPUS probe with a reproducible probe pressure, it would add another advantage to this imaging modality in comparison to TAUS imaging.

### 5.2. US Image Interpretation

The variation in US probe pressure applied by different operators may influence the displacement of the prostate and thus result in US image variation. However, also during interpretation of the images inter- and intraoperator variability can occur. This variability seems to be more present in operators with limited US imaging experience. For this reason, the importance of training has been emphasized by the American Association of Physicist in Medicine [72].

The inter- and intraoperator variability for different levels of expertise have been investigated in a few studies (Table 4). In these studies, the operators were asked to match a reference contour of the prostate to a newly acquired US image to determine the required setup shift during interfraction motion monitoring. Subsequently, differences in the performed matches were statistically examined.

The results reported by Fiandra et al. [32] show that the interuser variability decreases with growing TAUS imaging experience. The same holds for the intrauser variability during TPUS imaging, as reported by Pang et al. [33]. The operators that matched the images in the study of Van Der Meer et al. [17] received thorough training and scanning instructions. These operators seem to perform similarly to the operators with more than one year of experience of Fiandra et al. [32].

In Table 1, the results reported by Robinson et al. [16] regarding the differences in prostate localization between TAUS imaging and CT are listed. These results seem to confirm as well that more experience (clinical operator versus manufacturer representative) results in better agreement between the CT and TAUS based prostate locations.

In addition to providing training to the operators, making the system less prone to operator dependence could potentially reduce both inter- and intraoperator variability. In comparison with the Clarity system, the Clarity Autoscan system has already implemented several improvements to potentially reduce operator dependence. In particular, the mechanically swept probe could be attractive, since it minimizes the disadvantages of a manual sweep acquisition, such as the variance in probe pressure and sweeping motion. In addition, the probe is attached to a baseplate avoiding the need to hold it by hand and the operator is assisted to reproduce the earlier used probe pressure and setup by means of visual feedback.

Another approach to reduce operator dependence and potentially even allow less trained operators to acquire good-quality images was proposed by Camps et al. [73, 74]. In this work, the simulation CT scan of prostate cancer patients (currently almost always available for treatment planning purposes) was used to optimize the patient-specific US probe setup that would allow visualization of all the required anatomical structures with sufficient image quality. This helps to reduce the need for image interpretation during the acquisition and the operator variability in probe positioning.

## 6. Challenges 

Some challenges associated with the use of US imaging in the RT workflow have already been described in the previous sections, such as the inter- and intraoperator variability and the displacement of anatomical structures due to probe pressure. In this section, a number of other challenges associated with the implementation of US imaging in the prostate RT workflow are discussed.

### 6.1. Intrafraction US Imaging

The presence of the US probe in the radiation beam during the treatment can potentially cause dose delivery errors, which might influence the treatment outcome for the patient. Three possible solutions have been proposed in the literature for this problem. One option is to design the treatment plan in such a way that the US probe is completely avoided during the treatment [75]. Second, the radiation can be delivered through the probe, but it requires that the possible dose deviations are taken into account during the treatment planning process, as investigated by, for example, Bazalova-Carter et al. [76]. As a third solution, Schlosser and Hristov [77] designed a 4D radiolucent US probe with significantly less metal components close to the imaging field. This probe should produce a minimal interference with the radiation beam.

Martyn et al. [78] also investigated the effect of an US probe on the surface dose delivered to a phantom using a Monte Carlo study. In this study, a phantom was imaged using an Elekta Autoscan probe parallel to the radiation beam to mimic TAUS imaging, or perpendicular to the beam, to mimic TPUS imaging. It was shown that the presence of the probe in the TPUS configuration produces dose perturbations near the surface of the phantom, when there is overlap between the probe and the radiation field. However, the dose increase was of a similar order of magnitude as the one resulting from interfraction motion. In case no probe-field overlap occurred, the measured dosimetric effect was minimal. In the TAUS probe setup, instead, a dose increase near the surface of the phantom was measured and reported to be smaller than 5%.

Several studies (e.g., [75, 79–81]) also looked into the possibility of replacing a human operator handling the probe at the bedside with a robot. Schlosser et al. [75], for example, built a patient-safe robotic manipulator which could be used to control the pitch and pressure of a TAUS probe. To safely control the robot remotely from outside the LINAC room, a haptic device was added to the design. During the treatment delivery, the beam angles were restricted to prevent collision with the robotic hardware or the probe. The authors showed that the robotic system was able to image the prostate remotely. In addition, both the tracking ability of the US probe and the robot performance were not degraded during radiation beam operation. The use of such a robotic system could not only enable intrafraction TAUS imaging, but also potentially allow for an easier probe pressure and position reproduction using both TAUS and TPUS imaging.

### 6.2. Speed of Sound and Refraction Effects

Most clinical US systems work in pulse-echo mode, where the time of flight of the US pulses is used to infer the depth of the structures in the scanned tissues. This time of flight is calculated with the speed of sound (SOS) of the tissues traversed by the pulse. Different tissues have a different SOS. For example, adipose tissue typically has an SOS around 1450 m/s, while for connective tissue it is around 1600 m/s [82].

However, the US systems usually assume a fixed average SOS value of 1540 m/s for all human soft tissues [83]. This assumption may produce wrong quantitative estimates of organ boundary positions up to several millimeters. Fontanarosa et al. published multiple studies [84–87] in which CT scans were used to create SOS maps for correcting these aberrations. These corrections are essential to restore quantitative comparability with the reference simulation CT scan.

Not only does the usability of US imaging in the RT workflow rely on the acquisition and interpretation of the US images, but also the precision of the calibration procedure of the localization system and, associated with that, the precision that can be achieved while localizing the US probe in absolute coordinates in the simulation or treatment room are of importance. How well the US probe is localized influences the coregistration between, for example, the simulation CT scan and the reference US image, or two US images acquired at different time points.

The phantoms used in a calibration procedure are typically made of homogeneous tissue equivalents to avoid the SOS effects. In addition, refractions inside the phantom should not affect the calibration procedure. However, in the work of Ballhausen et al. [88] it has been shown that the calibration of a 3D US system can be affected by refraction of the sound waves at the phantom surface. Particularly when the probe was tilted during the calibration procedure this could result in a position difference of more than 0.5 mm.

Van der Meer et al. [89] simulated five different scenarios mimicking the errors that could occur when using the Clarity system for TAUS image guidance. These errors could be due to, for example, the above-mentioned inaccurate calibration, but also due to laser offsets or patient motion between the simulation CT and simulation US image acquisition. It has been shown that it is important to take SOS aberrations into account and to assess the matching of US and CT images. In case these images do not match, a manual correction could be performed, potentially introducing operator variability. In such a case, the authors recommend rescanning the patient to avoid problems during the dose delivery procedure.

Summarizing, it is important to take SOS aberrations into account while registering US images to another image modality. In addition, caution should be used while performing calibration and image acquisition, to avoid image matching issues.

### 6.3. Hypofractionation and Adaptive Radiotherapy

In current clinical practice, it is common to deliver the radiation dose to prostate cancer patients in multiple treatment fractions (even up to 45). It has been suggested that hypofractionation could result in the same or better outcomes for the prostate patients [90]. In a hypofractionation scheme, a higher dose per fraction is delivered to the patient in less treatment fractions. The treatment is then delivered over a shorter amount of time and with a total lower dose.

As the dose delivered per treatment fraction is higher and there are fewer fractions to potentially perform corrections or compensate for errors performed in the previous fractions, it is even more crucial to deliver the radiation correctly. Ricardi et al. [91] used the Clarity system in the treatment of intermediate risk prostate cancer patients treated with a hypofractionated schedule. It was shown that the hypofractionated schedule under US guidance was a safe and effective treatment approach with consistent biochemical control and a mild toxicity profile.

Patient immobilization during the treatment fraction is also an important aspect of the RT workflow. For this reason, a wide range of immobilization devices is available on the market, ranging from a simple leg immobilizer (Civco Medical Solutions, IA, USA) to vacuum cushions (e.g., Vac-Lok, Civco Medical Solutions, IA, USA) that can adapt to the body composition of the patient. Pang et al. [92] investigated the interfraction setup differences, patient satisfaction, and radiation therapist satisfaction regarding two immobilization devices: the traditionally used leg immobilizer and the Clarity Autoscan immobilization device. The results showed that the setup errors were smaller with the Clarity device and the patients were satisfied with the new device. The radiation therapist, though, had some issues with the weight and bulkiness of the new device.

ART aims at reducing or compensating for the effects of patient-specific treatment variation measured during the course of a radiotherapy treatment [93, 94] by adaptively modifying the treatment plan of the patient. This approach could be used to further improve the accuracy of radiation dose delivery. However, in current clinical practice, typically CT scans provide the electron-density information necessary for treatment planning and dose calculation. So, in case replanning proves necessary, one or multiple additional CT scans during the course of the treatment must be acquired. Not only does this result in extra radiation dose delivery to the patient, but also high costs are associated with the rather complex CT acquisition procedure.

Van Der Meer et al. [95] and Camps et al. [96] have investigated the feasibility of creating pseudo-CT scans of the pelvic region, based on combinations of rigid and deformable image registrations of TAUS images. These TAUS images acquired at simulation stage and during treatment stage were used to create a deformation field that represented the changes that occurred in tissue distribution between these two time points. The subsequent application of this deformation field on the simulation CT resulted in the creation of a pseudo-CT scan. It was shown that this pseudo-CT scan represents the anatomy of the patient at treatment stage better than the simulation CT. These results are promising and may lead to the ability to replan based on a pseudo-CT scan, instead of on a regular CT scan.

## 7. Conclusion

In this work, the recent relevant studies regarding the use of US imaging for guidance during the prostate EBRT workflow have been discussed. Several US based guidance systems have been introduced to the market in the last 15 years with varying success. TPUS imaging seems to overcome some of the issues associated with the limitations of TAUS imaging during intrafraction organ motion monitoring, such as displacement of the organs due to probe pressure and the interference with the radiation beam.

The studies that investigated TPUS imaging show promising results and, for this reason, we recommend the use of TPUS imaging during the US guided external beam radiotherapy workflow of prostate cancer patients. However, there are still several challenges to be addressed, which are associated with inter- and intraoperator variability during the acquisition of the images and the interpretation of these images. In addition, technical aspects of the US image modality, such as SOS aberrations and refractions should be investigated further to understand if these cause issues while using TPUS imaging for both inter- and intrafraction monitoring.

If a decrease in user variability and an increase of usability of the US guided EBRT systems can be achieved, this would potentially make the use of this approach more appealing to physicians and medical experts, in the end, resulting in smaller margins with less toxicities for prostate cancer patients undergoing EBRT.

## Figures and Tables

**Table 1:** Studies comparing TAUS or TPUS with other imaging techniques used for IGRT. The first column details the first author and year of publication. *∗* indicates that the paper was also included in [11, 12]. The second column details the used US technique: TAUS (Clarity) or TPUS (Clarity Autoscan). The third column indicates the parameters varied in the study. The fourth column indicates the image modality used for comparison with FM (fiducial marker) and EPI (electronic portal imaging). The columns five and six detail the number of patients and the number of acquired scans, respectively. Most studies reported the difference using mean ± standard deviation (SD) (column seven); however, one study used the error notation [13]. The square brackets indicate absolute values. The final column details the Bland-Altman Limits of Agreement (LoA) or, if this was not reported, the range of measured differences denoted between [ ].

First author	US	Parameters	Compared with	# pts	# US scans	Difference: mean ± SD [mm]			Bland-Altman/Range [mm]		
						AP	LR	SI	AP	LR	SI
Cury^*∗*^ (2006) [14]	TAUS	-	CT	10	30	−0.2 ± 1.6	0.2 ± 1.7	0.1 ± 1.4	-	-	-

Johnston^*∗*^ (2008) [15]	TAUS	Assisted segmentation	FM (EPI)	8	255	1.3 ± 6.6	0.9 ± 4.0	1.3 ± 5.1	−11.7–14.3	−7–8.8	−8.8–11.4
		Manual segmentation	FM (EPI)	7	181	2.1 ± 4.6	0.8 ± 3.5	0.2 ± 4.5	−6.8–11.1	−6.0–7.7	−8.7–9.0

Robinson^*∗*^ (2012) [16]	TAUS	(1) All datasets	CT	17	CT: 136 US: 272	10.3 ± 7.9			[1.3–61.4]		
		(2) Remove insufficient data	CT	-	US: 210	8.7 ± 4.9			[1.0–40.0]		
		(3) Review (2) manufacturer	CT	-	US: 153	7.4 ± 3.1			[1.8–17.1]		

Van Der Meer^*∗*^ (2013) [17]	TAUS	-	FM (EPI)	8	244	−2.3 ± 3.6	2.5 ± 4.0	0.6 ± 4.9	−9.3–4.7	−5.4–10.3	−8.9–10.2

Mayyas^*∗*^ (2013) [13]	TAUS	-	Bone (EPI)	27	1100	*μ*: −0.7 Σ: 2.4 *σ*: 3.4	*μ*: −0.5 Σ: 1.6 *σ*: 3.2	*μ*: −1.0 Σ: 2.4 *σ*: 3.6	*μ* = *mean error* Σ = *systematic error *[18] *σ* = *random error*		

Li (2015) [19]	TAUS	-	FM (CBCT)	6	78	0.0 ± 3.0	−0.2 ± 2.7	−1.9 ± 2.3	[−7.3–7.2]	[−5.6–6.9]	[−10.0–2.9]

Fargier-Voiron (2015) [20]	TAUS	Raw database	CBCT	25	284	2.8 ± 4.1	0.5 ± 3.3	−0.9 ± 4.2	−5.3–10.9	−5.9–6.9	−9.0–7.3
		Data corrected by mean US-CBCT difference of first 3 fractions	CBCT	25	284	−0.5 ± 3.9	0.3 ± 3.0	−1.0 ± 4.2	−8.1–7.1	−5.6–6.2	−7.7–7.3

Krengli (2016) [21]	TAUS	-	3D surface image	40	1318	−1.2 ± 4.9	−0.7 ± 5.0	−2.6 ± 6.4	[−25.8–18.0]	[−48.8–15.9]	[−22.5–22.1]

Richter (2016) [22]	TPUS	-	CBCT	10	150	[3.0 ± 2.4]	[2.7 ± 2.3]	[3.2 ± 2.7]	−7.1–8.2	−8–5	−9.4–6.5

Fargier-Voiron (2016) [23]	TPUS	With intrafraction motion	CBCT	12	357	1.9 ± 3.2	0.2 ± 2.6	0.7 ± 2.6	−4.3–8.1	−4.4–5.8	4.9–5.3
		No intrafraction motion	CBCT	12	357	2.8 ± 3	−0.1 ± 2.5	−0.3 ± 2.5	−3.2–8.8	−5–4.7	−5.1–4.6

Trivedi (2017) [24]	TPUS	-	FM (CT)	17	30	−0.06 ± 2.86	0.63 ± 3.27	−0.49 ± 3.49	[−4.55–7.52]	[−5.96–7.06]	[−6.70–6.78]

Li (2017) [25]	TPUS	-	FM (CBCT)	7	177	0.3 ± 1.7	0.0 ± 1.7	0.2 ± 2.0	[−4.2–5.5]	[−4.8–3.9]	[−4.5–5.7]

**Table 2:** Studies reporting on the use of TPUS imaging with the Clarity Autoscan system for intrafraction prostate motion monitoring. The first column details the first author and publication year. The second column details the used system, while the third and fourth column indicate the number of patients and scans examined, respectively. The fifth column contains the examined time intervals in seconds, while the final column details some results and conclusions.

First author	System	# pts	# US scans	Time [sec]	Results and conclusions
Baker (2016) [26]	TPUS	10	51	120–150	(i) Largest displacement (2.8 mm) in posterior direction (ii) Displacement insignificant during treatment time (iii) Displacement increases over time

Richardson (2017) [27]	TPUS	20	526	385	(i) Posterior motion seems most common (ii) 35% of patients displacement > 10 mm (iii) Duration of displacement varies considerably between patients

Guillet (2017) [28]	TPUS	10	330	140 (+120 setup)	(i) Largest movement in AP direction (ii) Dosimetric impact increases with treatment time duration
				290 (+120 setup)	

**Table 3:** Studies reporting on prostate displacement induced by probe pressure. The first column details the first author and publication year. Δ indicates that the specific study was mentioned in the previous review paper [11], but these specific results were not discussed. The second column details the used system, while the third column provides the imaging modality with which the prostate displacement was assessed. The fourth and fifth column specify the number of examined patients and the assessed scans, respectively. The prostate displacement in all directions is listed in column 6 with a indicating results per 1 mm probe shift and in the final column the displacement vector can be found.

First author	System	Assessed with	# pts	# US scans	Prostate displacement mean ± SD [mm]			Displacement vector mean ± SD [mm]
					AP	LR	SI	
Van Der Meer^Δ^ (2013) [17]	TAUS	Relative TAUS	13	376	0.7	−0.5	0.0	3.0
Fargier-Voiron^Δ^ (2014) [29]	TAUS	Relative TAUS	8	24	-	-	-	2.5 ± 1.2
Baker (2015) [30]	TAUS	TPUS	9	42	−0.1 ± 1.0	0.2 ± 0.7	−0.1 ± 0.8	1.3 ± 0.7
Li (2017) [31]	TPUS	Relative TPUS	10	16 series	0.07 ± 0.11^a^	0.04 ± 0.11^a^	0.42 ± 0.09^a^	2–4

**Table 4:** Studies reporting on the inter- and intraoperator variability of the Clarity system (TAUS) or the Clarity Autoscan system (TPUS). The first column details the first author and publication year. *∗* indicates that the detailed results were discussed in the review paper [11] as well, while Δ highlights that these specific results were not discussed, but the paper was included in the previous review. The second column specifies the used system, while the third and fourth column provide the number of compared operators and their experience, respectively. In the fifth column the number of examined patients is specified, while in the sixth column the number of matches made by the operators is detailed. The seventh column explains the metric that was used to quantify the intraoperator variability (column eight) and the interoperator variability (column nine).

First author	System	# operators	Experience	# pts	# matches	Metric	Intra			Inter		
							AP	LR	SI	AP	LR	SI
Van der Meer^*∗*^ (2013) [17]	TAUS	2 (intra) 3 (inter)	-	13	817	SD (mm)	0.7	0.8	1	1.4	1.3	1.8

Fiandra^Δ^ (2014) [32]	TAUS	2	Expert (>5 years)	10	60	Mean ± SD of operator Δ (mm)	-	-	-	−0.1 ± 1.4	−0.4 ± 1.2	0.1 ± 1.3
		5	>1 year	10	150	Root mean square error with respect to expert [mm]	-	-	-	2.1 ± 2.1	1.3 ± 1.7	1.7 ± 1.7
		4	<1 year	10	120		-	-	-	3.1 ± 2.7	2.7 ± 2.7	3.2 ± 3.2

Pang (2017) [33]	TPUS	7	All operators	10	70	Δ between operator and total group median [mm] in all directions	≤2 in 93.3% of the time			≤2 in 93.8% of the time		
		5	9–16 months	10	50		≤2 in 96.7% of the time			-		
		2	4–9 months	10	20		≤2 in 60% and 80% of the time			-		

## Abbreviations

AP: Anterior-posterior
ART: Adaptive radiotherapy
BAT: B-mode acquisition and targeting
CBCT: Cone beam computed tomography
CT: Computed tomography
EBRT: External beam radiotherapy
EPI: Electronic portal imaging
FM: Fiducial marker
IGRT: Image guided radiotherapy
IMRT: Intensity-modulated radiotherapy
kV: Kilovolt
LINAC: Linear accelerator
LoA: Limits of agreement
LR: Left-right
MRI: Magnetic resonance imaging
OAR: Organ at risk
RT: Radiotherapy
SD: Standard deviation
SI: Superior-inferior
SOS: Speed of sound
TAUS: Transabdominal ultrasound
TPUS: Transperineal ultrasound
TRUS: Transrectal ultrasound
US: Ultrasound
VMAT: Volumetric modulated arc therapy
2D: Two-dimensional
3D: Three-dimensional.

## References

[1] Fitzmaurice C., Allen C., Barber R. M. (2017). Global, regional, and national cancer incidence, mortality, years of life lost, years lived with disability, and disability-adjusted life-years for 32 cancer groups, 1990 to 2015: a systematic analysis for the global burden of disease study. *JAMA Oncology*.

[2] Torre L. A., Siegel R. L., Ward E. M., Jemal A. (2016). Global cancer incidence and mortality rates and trends—an update. *Cancer Epidemiology, Biomarkers & Prevention*.

[3] Vanneste B. G. L., Van Limbergen E. J., Van Lin E. N., Van Roermund J. G. H., Lambin P. (2016). Prostate Cancer Radiation Therapy: What Do Clinicians Have to Know?. *BioMed Research International*.

[4] Zelefsky M. J., Kollmeier M., Cox B. (2012). Improved clinical outcomes with high-dose image guided radiotherapy compared with non-IGRT for the treatment of clinically localized prostate cancer. *International Journal of Radiation Oncology • Biology • Physics*.

[5] Sveistrup J., af Rosenschöld P. M., Deasy J. O. (2014). Improvement in toxicity in high risk prostate cancer patients treated with image-guided intensity-modulated radiotherapy compared to 3D conformal radiotherapy without daily image guidance. *Journal of Radiation Oncology*.

[6] van der Heide U. A., Kotte A. N. T. J., Dehnad H., Hofman P., Lagenijk J. J. W., van Vulpen M. (2007). Analysis of fiducial marker-based position verification in the external beam radiotherapy of patients with prostate cancer. *Radiotherapy & Oncology*.

[7] Lagendijk J. J. W., Raaymakers B. W., van Vulpen M. (2014). The Magnetic Resonance Imaging-Linac System. *Seminars in Radiation Oncology*.

[8] Foster R. D., Solberg T. D., Li H. S. (2010). Comparison of transabdominal ultrasound and electromagnetic transponders for prostate localization. *Journal of Applied Clinical Medical Physics*.

[9] Fung A. Y. C., Ayyangar K. M., Djajaputra D., Nehru R. M., Enke C. A. (2006). Ultrasound-based guidance of intensity-modulated radiation therapy. *Medical Dosimetry*.

[10] Shen D., Zhan Y., Davatzikos C. (2003). Segmentation of prostate boundaries from ultrasound images using statistical shape model. *IEEE Transactions on Medical Imaging*.

[11] Fontanarosa D., Van Der Meer S., Bamber J., Harris E., O'Shea T., Verhaegen F. (2015). Review of ultrasound image guidance in external beam radiotherapy: I. Treatment planning and inter-fraction motion management. *Physics in Medicine and Biology*.

[12] O'Shea T., Bamber J., Fontanarosa D., Van Der Meer S., Verhaegen F., Harris E. (2016). Review of ultrasound image guidance in external beam radiotherapy part II: Intra-fraction motion management and novel applications. *Physics in Medicine and Biology*.

[13] Mayyas E., Chetty I. J., Chetvertkov M. (2013). Evaluation of multiple image-based modalities for image-guided radiation therapy (IGRT) of prostate carcinoma: A prospective study. *Medical Physics*.

[14] Cury F. L. B., Shenouda G., Souhami L. (2006). Ultrasound-based image guided radiotherapy for prostate cancer-comparison of cross-modality and intramodality methods for daily localization during external beam radiotherapy. *International Journal of Radiation Oncology • Biology • Physics*.

[15] Johnston H., Hilts M., Beckham W., Berthelet E. (2008). 3D ultrasound for prostate localization in radiation therapy: A comparison with implanted fiducial markers. *Medical Physics*.

[16] Robinson D., Liu D., Steciw S. (2012). An evaluation of the clarity 3D ultrasound system for prostate localization. *Journal of Applied Clinical Medical Physics*.

[17] Van Der Meer S., Bloemen-Van Gurp E., Hermans J. (2013). Critical assessment of intramodality 3D ultrasound imaging for prostate IGRT compared to fiducial markers. *Medical Physics*.

[18] van Herk M. (2004). Errors and margins in radiotherapy. *Seminars in Radiation Oncology*.

[19] Li M., Ballhausen H., Hegemann N.-S. (2015). A comparative assessment of prostate positioning guided by three-dimensional ultrasound and cone beam CT. *Journal of Radiation Oncology*.

[20] Fargier-Voiron M., Presles B., Pommier P. (2015). Ultrasound versus Cone-beam CT image-guided radiotherapy for prostate and post-prostatectomy pretreatment localization. *Physica Medica*.

[21] Krengli M., Loi G., Pisani C. (2016). Three-dimensional surface and ultrasound imaging for daily IGRT of prostate cancer. *Journal of Radiation Oncology*.

[22] Richter A., Polat B., Lawrenz I. (2016). Initial results for patient setup verification using transperineal ultrasound and cone beam CT in external beam radiation therapy of prostate cancer. *Journal of Radiation Oncology*.

[23] Fargier-Voiron M., Presles B., Pommier P. (2016). Evaluation of a new transperineal ultrasound probe for inter-fraction image-guidance for definitive and post-operative prostate cancer radiotherapy. *Physica Medica*.

[24] Trivedi A., Ashikaga T., Hard D. (2017). Development of 3-dimensional transperineal ultrasound for image guided radiation therapy of the prostate: Early evaluations of feasibility and use for inter- and intrafractional prostate localization. *Practical Radiation Oncology*.

[25] Li M., Ballhausen H., Hegemann N.-S. (2017). Comparison of prostate positioning guided by three-dimensional transperineal ultrasound and cone beam CT. *Strahlentherapie und Onkologie*.

[26] Baker M., Behrens C. F. (2016). Determining intrafractional prostate motion using four dimensional ultrasound system. *BMC Cancer*.

[27] Richardson A. K., Jacobs P. (2017). Intrafraction monitoring of prostate motion during radiotherapy using the Clarity® Autoscan Transperineal Ultrasound (TPUS) system. *Radiography*.

[28] Guillet L., Fargier-Voiron M., Sarrut D., Biston M.-C. (2015). Evaluation of intrafraction motions with a transperineal ultrasound imaging system: dosimetric impact for prostate cancer. *Physica Medica: European Journal of Medical Physics*.

[29] Fargier-Voiron M., Presles B., Pommier P. (2014). Impact of probe pressure variability on prostate localization for ultrasound-based image-guided radiotherapy. *Radiotherapy & Oncology*.

[30] Baker M., Behrens C. F. (2015). Prostate displacement during transabdominal ultrasound image-guided radiotherapy assessed by real-time four-dimensional transperineal monitoring. *Acta Oncologica*.

[31] Li M., Hegemann N.-S., Manapov F. (2017). Prefraction displacement and intrafraction drift of the prostate due to perineal ultrasound probe pressure. *Strahlentherapie und Onkologie*.

[32] Fiandra C., Guarneri A., Muñoz F. (2014). Impact of the observers' experience on daily prostate localization accuracy in ultrasound-based IGRT with the Clarity platform. *Journal of Applied Clinical Medical Physics*.

[33] Pang E. P. P., Knight K., Baird M., Tuan J. K. L. (2017). Inter-and intra-observer variation of patient setup shifts derived using the 4D TPUS Clarity system for prostate radiotherapy. *Biomedical Physics & Engineering Express*.

[34] Lachaine M., Falco T. (2013). Intrafractional prostate motion management with the Clarity Autoscan system. *Medical Physics International*.

[35] Parker C. C., Damyanovich A., Haycocks T., Haider M., Bayley A., Catton C. N. (2003). Magnetic resonance imaging in the radiation treatment planning of localized prostate cancer using intra-prostatic fiducial markers for computed tomography co-registration. *Radiotherapy & Oncology*.

[36] Milosevic M., Voruganti S., Blend R. (1998). Magnetic resonance imaging (MRI) for localization of the prostatic apex: comparison to computed tomography (CT) and urethrography. *Radiotherapy & Oncology*.

[37] Rasch C., Barillot I., Remeijer P., Touw A., van Herk M., Lebesque J. V. (1999). Definition of the prostate in CT and MRI: a multi-observer study. *International Journal of Radiation Oncology • Biology • Physics*.

[38] Horsley P. J., Aherne N. J., Edwards G. V. (2015). Planning magnetic resonance imaging for prostate cancer intensity-modulated radiation therapy: impact on target volumes, radiotherapy dose and androgen deprivation administration. *Asia-Pacific Journal of Clinical Oncology*.

[39] Bentel G. (1999). *Patient Positioning and Immobilization in Radiation Oncology*.

[40] O'neill A. G. M., Jain S., Hounsell A. R., O'sullivan J. M. (2016). Fiducial marker guided prostate radiotherapy: A review. *British Journal of Radiology*.

[41] Oldham M., Létourneau D., Watt L. (2005). Cone-beam-CT guided radiation therapy: a model for on-line application. *Radiotherapy & Oncology*.

[42] Langen K. M., Jones D. T. L. (2001). Organ motion and its management. *International Journal of Radiation Oncology • Biology • Physics*.

[43] Hill C. R., Bamber J. C., ter Haar G. R. (2005). Preface. *Physical Principles of Medical Ultrasonics*.

[44] Bushberg J. T. (2002). *The Essential Physics of Medical Imaging*.

[45] Aarnink R. G., Beerlage H. P., De La Rosette J. J. M. C. H., Debruyne F. M. J., Wijkstra H. (1998). Transrectal ultrasound of the prostate: Innovations and future applications. *The Journal of Urology*.

[46] Gill S., Li J., Thomas J. (2012). Patient-reported complications from fiducial marker implantation for prostate image-guided radiotherapy. *British Journal of Radiology*.

[47] Langenhuijsen J. F., van Lin E. N. J. T., Kiemeney L. A. (2007). Ultrasound-guided transrectal implantation of gold markers for prostate localization during external beam radiotherapy: complication rate and risk factors. *International Journal of Radiation Oncology • Biology • Physics*.

[48] Huang Foen Chung J. W. N. C., De Vries S. H., Raaijmakers R., Postma R., Bosch J. L. H. R., Van Mastrigt R. (2004). Prostate volume ultrasonography: The influence of transabdominal versus transrectal approach, device type and operator. *European Urology*.

[49] Uppot R. N., Sahani D. V., Hahn P. F., Kalra M. K., Saini S. S., Mueller P. R. (2006). Effect of obesity on image quality: Fifteen-year longitudinal study for evaluation of dictated radiology reports. *Radiology*.

[50] Uppot R. N. (2007). Impact of Obesity on Radiology. *Radiologic Clinics of North America*.

[51] Salter B. J., Szegedi M., Boehm C. (2017). Comparison of 2 transabdominal ultrasound image guidance techniques for prostate and prostatic fossa radiation therapy. *Practical Radiation Oncology*.

[52] Griffiths K. A., Ly L. P., Jin B., Chan L., Handelsman D. J. (2007). Transperineal Ultrasound for Measurement of Prostate Volume: Validation Against Transrectal Ultrasound. *The Journal of Urology*.

[53] Shinohara K., Roach M. (2008). Technique for Implantation of Fiducial Markers in the Prostate. *Urology*.

[54] Iğdem Ş., Akpinar H., Alço G., Ağaçayak F., Turkan S., Okkan S. (2009). Implantation of fiducial markers for image guidance in prostate radiotherapy: Patient-reported toxicity. *British Journal of Radiology*.

[55] Loh J., Baker K., Sridharan S. (2015). Infections after fiducial marker implantation for prostate radiotherapy: Are we underestimating the risks?. *Journal of Radiation Oncology*.

[56] Kupelian P. A., Willoughby T. R., Meeks S. L. (2005). Intraprostatic fiducials for localization of the prostate gland: Monitoring intermarker distances during radiation therapy to test for marker stability. *International Journal of Radiation Oncology • Biology • Physics*.

[57] Chow J. C. L., Grigorov G. N. (2005). Dose measurements near a non-radioactive gold seed using radiographic film. *Physics in Medicine and Biology*.

[58] Perks J. R., Lehmann J., Chen A. M., Yang C. C., Stern R. L., Purdy J. A. (2008). Comparison of peripheral dose from image-guided radiation therapy (IGRT) using kV cone beam CT to intensity-modulated radiation therapy (IMRT). *Radiotherapy & Oncology*.

[59] Tas B., Durmus I. F., Ozturk S. T. (2016). Image guided radiotherapy (igrt) comparison between cone beam ct and ultrasound system for prostate cancer. *Universal Journal of Physics and Application*.

[60] Martin Bland J., Altman D. (1986). Statistical methods for assessing agreement between two methods of clinical measurement. *The Lancet*.

[61] Meijer G. J., de Klerk J., Bzdusek K. (2008). What CTV-to-PTV Margins Should Be Applied for Prostate Irradiation? Four-Dimensional Quantitative Assessment Using Model-Based Deformable Image Registration Techniques. *International Journal of Radiation Oncology • Biology • Physics*.

[62] Kupelian P., Willoughby T., Mahadevan A. (2007). Multi-institutional clinical experience with the Calypso System in localization and continuous, real-time monitoring of the prostate gland during external radiotherapy. *International Journal of Radiation Oncology • Biology • Physics*.

[63] Balter J. M., Wright J. N., Newell L. J. (2005). Accuracy of a wireless localization system for radiotherapy. *International Journal of Radiation Oncology • Biology • Physics*.

[64] Abramowitz M. C., Bossart E., Flook R. (2012). Noninvasive real-time prostate tracking using a transperineal ultrasound approach. *International Journal of Radiation Oncology, Biology, Physics*.

[65] Yu A. S., Najafi M., Hristov D. H., Phillips T. (2017). Intrafractional tracking accuracy of a transperineal ultrasound image guidance system for prostate radiotherapy. *Technology in Cancer Research & Treatment*.

[66] Ballhausen H., Li M., Hegemann N.-S., Ganswindt U., Belka C. (2015). Intra-fraction motion of the prostate is a random walk. *Physics in Medicine and Biology*.

[67] Ballhausen H., Reiner M., Kantz S., Belka C., Söhn M. (2013). The random walk model of intrafraction movement. *Physics in Medicine and Biology*.

[68] Hilman S., Smith R., Masson S. (2017). Implementation of a daily transperineal ultrasound system as image-guided radiotherapy for prostate cancer. *Clinical Oncology*.

[69] Hilman S., Jacobs P. (2017). Image-guided radiotherapy for prostate cancer using transperineal ultrasound. *RAD Magazine*.

[70] Mantel F., Richter A., Groh C. (2016). Changes in penile bulb dose when using the Clarity transperineal ultrasound probe: A planning study. *Practical Radiation Oncology*.

[71] Mangar S. A., Sydes M. R., Tucker H. L. (2006). Evaluating the relationship between erectile dysfunction and dose received by the penile bulb: Using data from a randomised controlled trial of conformal radiotherapy in prostate cancer (MRC RT01, ISRCTN47772397). *Radiotherapy & Oncology*.

[72] Molloy J. A., Chan G., Markovic A. (2011). Quality assurance of U.S.-guided external beam radiotherapy for prostate cancer: Report of AAPM Task Group 154. *Medical Physics*.

[73] Camps S. M., Verhaegen F., Paiva Fonseca G., De With P. H. N., Fontanarosa D. Automatic transperineal ultrasound probe positioning based on CT scan for image guided radiotherapy.

[74] Camps S., Verhaegen F., de With P. H. N., Fontanarosa D. CT Scan Based Prostate Cancer Patient-Specific Transperineal Ultrasound Probe Setups for Image Guided Radiotherapy.

[75] Schlosser J., Salisbury K., Hristov D. (2010). Telerobotic system concept for real-time soft-tissue imaging during radiotherapy beam delivery. *Medical Physics*.

[76] Bazalova-Carter M., Schlosser J., Chen J., Hristov D. (2015). Monte Carlo modeling of ultrasound probes for image guided radiotherapy. *Medical Physics*.

[77] Schlosser J., Hristov D. (2016). Radiolucent 4D Ultrasound Imaging: System Design and Application to Radiotherapy Guidance. *IEEE Transactions on Medical Imaging*.

[78] Martyn M., O'Shea T. P., Harris E., Bamber J., Gilroy S., Foley M. J. (2017). A Monte Carlo study of the effect of an ultrasound transducer on surface dose during intrafraction motion imaging for external beam radiation therapy. *Medical Physics*.

[79] Bell M. A. L., Sen H. T., Iordachita I., Kazanzides P., Wong J. (2014). In vivo reproducibility of robotic probe placement for a novel ultrasound-guided radiation therapy system. *Journal of Medical Imaging*.

[80] Şen H. T., Bell M. A. L., Zhang Y. System integration and preliminary in-vivo experiments of a robot for ultrasound guidance and monitoring during radiotherapy.

[81] Gerlach S., Kuhlemann I., Jauer P. (2017). Robotic ultrasound-guided SBRT of the prostate: feasibility with respect to plan quality. *International Journal for Computer Assisted Radiology and Surgery*.

[82] Mast T. D. (2000). Empirical relationships between acoustic parameters in human soft tissues. *Acoustic Research Letters Online*.

[83] Wells P. N. T.

[84] Fontanarosa D., Van Der Meer S., Harris E., Verhaegen F. (2011). A CT based correction method for speed of sound aberration for ultrasound based image guided radiotherapy. *Medical Physics*.

[85] Fontanarosa D., Van Der Meer S., Bloemen-Van Gurp E., Stroian G., Verhaegen F. (2012). Magnitude of speed of sound aberration corrections for ultrasound image guided radiotherapy for prostate and other anatomical sites. *Medical Physics*.

[86] Fontanarosa D., Van Der Meer S., Verhaegen F. (2012). On the significance of density-induced speed of sound variations on US-guided radiotherapy. *Medical Physics*.

[87] Fontanarosa D., Pesente S., Pascoli F., Ermacora D., Rumeileh I. A., Verhaegen F. (2013). A speed of sound aberration correction algorithm for curvilinear ultrasound transducers in ultrasound-based image-guided radiotherapy.. *Physics in Medicine and Biology*.

[88] Ballhausen H., Ballhausen B. D., Lachaine M. (2015). Surface refraction of sound waves affects calibration of three-dimensional ultrasound. *Journal of Radiation Oncology*.

[89] van der Meer S., Seravalli E., Fontanarosa D., Bloemen-van Gurp E. J., Verhaegen F. (2016). Consequences of Intermodality Registration Errors for Intramodality 3D Ultrasound IGRT. *Technology in Cancer Research & Treatment*.

[90] Dearnaley D., Syndikus I., Mossop H. (2016). Conventional versus hypofractionated high-dose intensity-modulated radiotherapy for prostate cancer: 5-year outcomes of the randomised, non-inferiority, phase 3 CHHiP trial. *The Lancet Oncology*.

[91] Ricardi U., Franco P., Munoz F. (2015). Three-dimensional ultrasound-based image-guided hypofractionated radiotherapy for intermediate-risk prostate cancer: Results of a consecutive case series. *Cancer Investigation*.

[92] Pang E. P. P., Knight K., Baird M., Loh J. M. Q., Boo A. H. S., Tuan J. K. L. (2017). A comparison of interfraction setup error, patient comfort, and therapist acceptance for 2 different prostate radiation therapy immobilization devices. *Advances in Radiation Oncology*.

[93] Yan D., Vicini F., Wong J., Martinez A. (1997). Adaptive radiation therapy. *Physics in Medicine and Biology*.

[94] Ghilezan M., Yan D., Martinez A. (2010). Adaptive Radiation Therapy for Prostate Cancer. *Seminars in Radiation Oncology*.

[95] Van Der Meer S., Camps S. M., Van Elmpt W. J. C. (2016). Simulation of pseudo-CT images based on deformable image registration of ultrasound images: A proof of concept for transabdominal ultrasound imaging of the prostate during radiotherapy. *Medical Physics*.

[96] Camps S., van der S., Meer F., Fontanarosa D. (2016). Various approaches for pseudo-CT scan creation based on ultrasound to ultrasound deformable image registration between different treatment time points for radiotherapy treatment plan adaptation in prostate cancer patients. *Biomedical Physics & Engineering Express*.

